# Incidence and risk factors of second primary cancer after the initial primary human papillomavirus related neoplasms

**DOI:** 10.1002/mco2.43

**Published:** 2020-12-03

**Authors:** Jiayi Shen, Huaqiang Zhou, Jiaqing Liu, Zhonghan Zhang, Wenfeng Fang, Yunpeng Yang, Shaodong Hong, Wei Xian, Yuxiang Ma, Ting Zhou, Yaxiong Zhang, Hongyun Zhao, Yan Huang, Li Zhang

**Affiliations:** ^1^ Department of Medical Oncology, Sun Yat‐sen University Cancer Center, State Key Laboratory of Oncology in South China Collaborative Innovation Center for Cancer Medicine Guangzhou China; ^2^ Zhongshan School of Medicine Sun Yat‐sen University Guangzhou China; ^3^ Department of Clinical Research, Sun Yat‐sen University Cancer Center, State Key Laboratory of Oncology in South China Collaborative Innovation Center for Cancer Medicine Guangzhou China

**Keywords:** human papillomavirus, risk, second primary cancer

## Abstract

Comprehensive studies in second primary cancer (SPC) after the initial primary human papillomavirus (HPV)‐related cancer still remain warranted. We aimed to analyze the incidence and risk factors of SPC after HPV‐related cancer. We identified 86 790 patients diagnosed with initial primary HPV‐related cancer between 1973 and 2010 in the SEER database. Standardized incidence ratio (SIR) and cumulative incidence were calculated to assess the risk of SPC after HPV‐related cancer. The SIR of SPC after HPV‐related cancer was 1.60 (95% confidence interval [CI], 1.55‐1.65) for male and 1.25 (95% CI, 1.22‐1.28) for female. SIR of second primary HPV‐related cancer (7.39 [95% CI, 6.26‐8.68] male and 4.35 [95% CI, 4.04‐4.67] female) was significantly higher than that of HPV‐unrelated cancer (1.54 [95% CI, 1.49‐1.60] male and 1.16 [95% CI, 1.13‐1.19] female). The 5‐year cumulative incidence of SPC was 7.22% (95% CI, 6.89‐7.55%) for male and 3.72% (95% CI, 3.58‐3.88%) for female. Risk factors for SPC included being married and having initial primary cancer (IPC) diagnosed at earlier stage for both genders, and IPC diagnosed at older age as well as surgery performed for female. Patients diagnosed with HPV‐related cancer are more likely to develop another primary cancer, compared with the age‐specific reference population.

## Graphical abstract

Our study performed a systematic estimation of the incidence of second primary cancer in HPV‐related cancer patients. This figure displayed the distribution of second primary cancer sites after the specific initial primary HPV‐related cancer. The result may provide some accordance for the surveillance of second primary cancer after initial primary HPV‐related cancer.

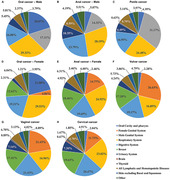

## 1 INTRODUCTION

Human papillomavirus (HPV) infection is a common viral infection, with its relatively high prevalence (11% in oral cavity, 26.8% for females in genital organs, 14% for females in anus, 45.2% for males in genital organs, and 16% for males in anus).^1^, ^2^, ^3^, ^4^, ^5^ Many types of HPV have been classified as Group 1 carcinogen by the International Agency for Research on Cancer (IARC). Specifically, HPV types 16, 18, 31, 33, 35, 39, 45, 51, 52, 56, 58, 59, and 66 have been classified as high‐risk HPV. HPV is a carcinogen in some cancers, with base of tongue (84% of the cases infected with HPV), tonsillar (51%), oropharyngeal (44.1%), Waldeyer's ring (32.9%), anal (84.3%), vulvar (40.4%), vaginal (69.9%), cervical (70%), and penile (47.9%) cancers included.^6^, ^7^, ^8^, ^9^, ^10^, ^11^, ^12^, ^13^, ^14^ We grouped cancers listed above as HPV‐related cancer and the rest as HPV‐unrelated cancer. According to the study by de Martel et al, HPV infection is responsible for 4.5% (630 000 new cases per year) of all cancer cases worldwide. The attributable fraction is <3% in some regions, while others are >20%.^15^ Despite the burden of HPV‐related cancer, we have seen improvement in the prognosis of HPV‐related cancer over time, due to the more advanced treatment and earlier detection.^16^, ^17^, ^18^, ^19^, ^20^, ^21^ Moreover, the improved survival observed can be the consequence of the elevation of proportion of HPV‐positive cases in HPV‐related cancers.^9^, ^22^, ^23^ HPV‐negative cervical cancer was found to be correlated with advanced stages at diagnosis, higher prevalence of lymph node metastases, and shorter progression‐free survival than HPV‐positive cervical cancer.^24^ Improved survival from the initial primary cancer (IPC) means an elevated risk of second primary cancer (SPC).^25^ The investigation into SPC after HPV‐related cancer is urgent and necessary for the surveillance strategies, based on the high prevalence of HPV‐related cancer and the improved survival.

There have been some reports of the incidence of SPC after some of the HPV‐related cancers. Standardized incidence ratio (SIR) after oral, oropharyngeal, anal, and cervical cancer was 2.82, 2.99, 1.41, and 1.56, respectively.^26^, ^27^, ^28^ However, in the light of the strong carcinogenic effect of HPV, HPV‐related cancer should be classified as a whole when considering SPC, to observe whether there are specific patterns of the occurrence of SPC. Here, we aimed to analyze the incidence and risk factors of SPC after initial primary HPV‐related cancer.

## 2 METHODS

The Surveillance, Epidemiology, and End Results (SEER) program has been collecting demographic and clinical characteristics and follow up of cancer patients in America since 1973, with its registries covering approximately 34.6% of the United States population currently. In this study, we acquired data from SEER 9, a database in SEER*Stat software version 8.3.5 containing information on cancer patients from nine different registers.

The patient cohort to be observed in this study included those who were diagnosed with HPV‐related cancer as their IPC between 1973 and 2010. IPC was defined as the first recorded primary malignant tumor in the SEER system. HPV‐related cancer was selected according to the International Classification of Diseases for Oncology, 3rd Edition (ICD‐O‐3) Site Codes, which included cancer in base of tongue (C01.9), tonsillar cancer (C02.4, C09), oropharyngeal cancer (C10), Waldeyer's ring cancer (C14.2), anal canal cancer (C21), vulvar cancer (C51), vaginal cancer (C52.9), cervical cancer (C53), and penile cancer (C60).^6^, ^7^, ^8^, ^9^ Those diagnosed with HPV‐related cancer before the age of 18 were not involved in this study. Records with death certificate or autopsy only were excluded. Overall, 86 790 patients diagnosed with initial primary HPV‐related cancer were included.

SPC was defined as the second primary malignant tumor recorded in the SEER system diagnosed between January 1973 and December 2015 and with a latency of over or equal to 6 months.^29^ The years of 2010 and 2015 were selected as the last years for the diagnosis of initial primary HPV‐related cancer and SPC, respectively, to ensure that all patients included were followed up for at least 5 years.

The SIR of SPC is defined as the observed number of SPC cases in the study cohort divided by the expected number of cases computed using age‐specific rates from a reference population, weighted according to the age structure of the study population.^30^ The calculation of SIR was done via the MP‐SIR session in SEER software. SIR of SPC after the diagnosis of HPV‐related cancer and HPV‐unrelated cancer were calculated.

In this study, death is regarded as a competing event with SPC. Cumulative incidence analysis was completed under consideration of competing risk. To identify the risk factors for SPC after the initial primary HPV‐related cancer, we conducted a multivariable subdistribution hazard regression, regarding death as the competing event with SPC.^31^ Age, race, marital status, tumor, node, and metastasis (TNM) stage, and surgery were considered to be potential risk factors for SPC. When conducting subdistribution hazard regression, patient cohort merely included those who were diagnosed with HPV‐related cancer as their IPC between 2004 and 2010, for the reason that the TNM stage (sixth edition) is only available since 2004. Correspondingly, SPC was defined as SPC records in the SEER system diagnosed between January 2004 and December 2015.

All the results were presented by gender, except when the IPC was genital cancer, to avoid unexpected bias. Both the cumulative incidence analysis and the subdistribution hazard regression were performed using R version 3.5.0 (Institute for Statistics and Mathematics, Vienna, Austria; www.r-project.org). Statistical significance was set at two‐sided *P* < .05.

## 3 RESULTS

### 3.1 Incidence of SPC

**FIGURE 1:**
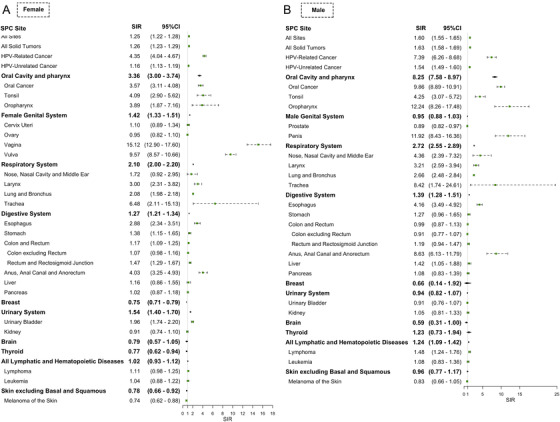
Subsite‐specific standardized incidence ratio (SIR) of second primary cancer (SPC) after the diagnosis of the initial primary human papillomavirus (HPV)‐related cancer stratified by sex. Subsite‐specific SIRs of developing SPC among survivors of initial primary HPV‐related cancer were shown. A, The SIRs of second primary oral cavity and pharynx, genital system, respiratory system, digestive system, breast, urinary system, brain, thyroid, hematopoietic, and skin cancer after initial primary HPV‐related cancer among females. B, The SIRs of second primary oral cavity and pharynx, genital system, respiratory system, digestive system, breast, urinary system, brain, thyroid, hematopoietic, and skin cancer after initial primary HPV‐unrelated cancer among men. The green dots represent SIR, and error bars represent 95% confidence interval. The vertical grey line indicates an SIR of 1.

The SIR of SPC after HPV‐related cancer was 1.60 (95% confidence interval [CI], 1.55‐1.65) for male and 1.25 (95% CI, 1.22‐1.28) for female (Figure 1). Moreover, SIR of second primary HPV‐related cancer (7.39 [95% CI, 6.26‐8.68] male and 4.35 [95% CI, 4.04‐4.67] female) was obviously higher than that of second primary HPV‐unrelated cancer (1.54 [95% CI, 1.49‐1.60] male and 1.16 [95% CI, 1.13‐1.19] female). When SPC site was limited to every single HPV‐related site, a consistent elevation of SIR was observed. However, notably, for female patients, SIR of SPC in cervix uteri (1.10 [95% CI, 0.89‐1.34]) was similar to that of SPC in all sites (1.25 [95% CI, 1.22‐1.28]). There has been insufficient evidence for the role of HPV in several cancers, which were considered as potentially HPV‐related cancers in this study, with esophagus, larynx, nose and nasal sinus, lung, colon and rectum, breast, prostate, and urinary bladder included.^32^, ^33^, ^34^, ^35^, ^36^, ^37^, ^38^, ^39^ We also observed an increase in SIR of SPC among some of the potentially HPV‐related sites, with esophagus (4.16 [95% CI, 3.49‐4.93] male and 2.88 [95% CI, 2.34‐3.51] female), larynx (3.21 [95% CI, 2.59‐3.94] male and 3.00 [95% CI, 2.31‐3.82] female), nose and nasal sinus (4.36 [95% CI, 2.39‐7.32] for male only), lung and bronchus (2.66 [95% CI, 2.48‐2.84] male and 2.08 [95% CI, 1.98‐2.18] female), trachea (8.42 [95% CI, 1.74‐24.61] male and 6.48 [95% CI, 2.11‐15.13] female), and urinary bladder (1.96 [95% CI, 1.74‐2.20] for female only) included.

**FIGURE 2:**
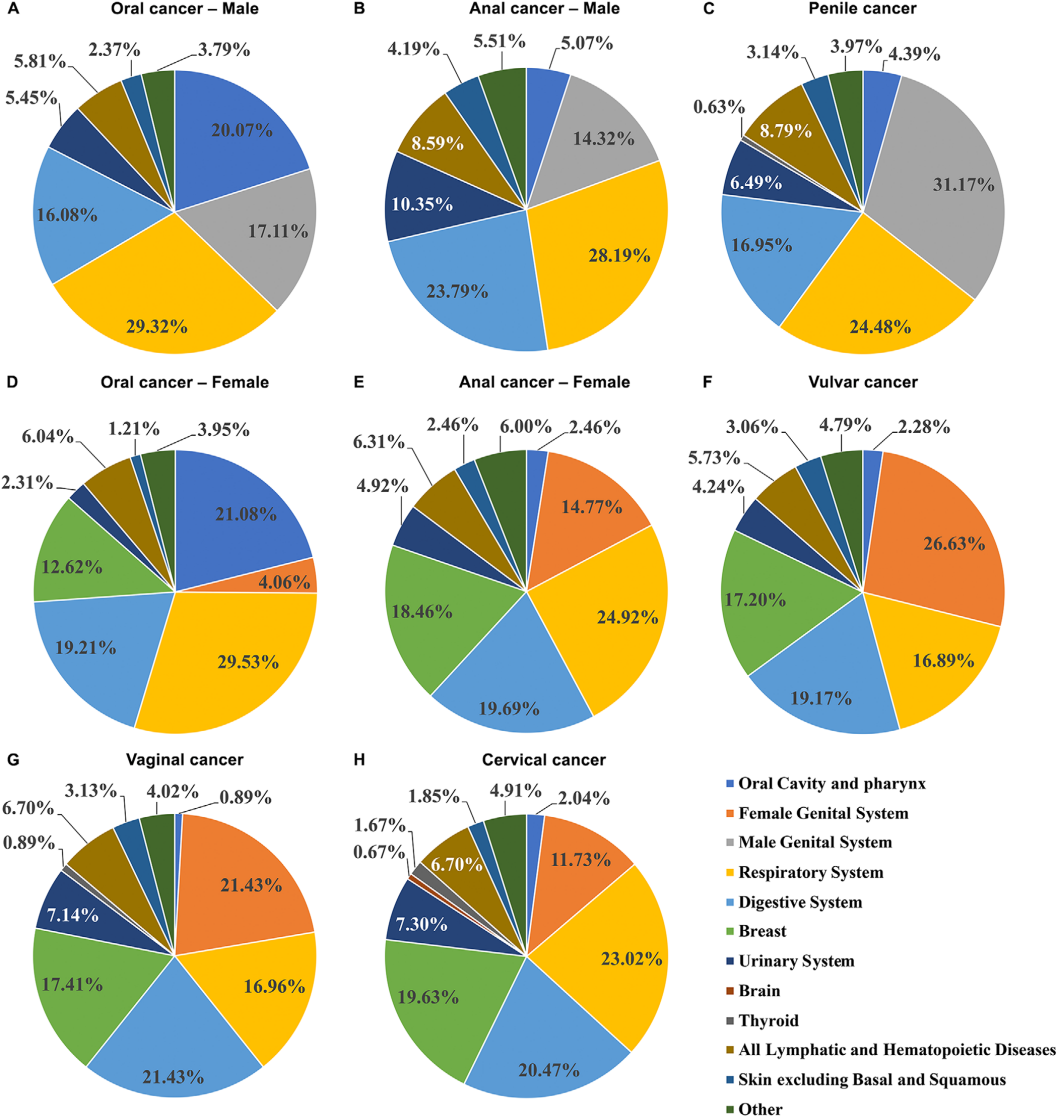
Distribution of second primary cancer (SPC) sites of initial primary human papillomavirus (HPV)‐related cancer (subsite‐specific). Shown are distribution of SPC sites of initial primary HPV‐related cancer. A, Oral cancer ‐ male. B, Anal cancer ‐ male. C, Penile cancer. D, Oral cancer ‐ female. E, Anal cancer ‐ female. F, Vulvar cancer. G, Vaginal cancer. H, Cervical cancer. The percentages displayed were calculated by dividing the number of observed cases in the SPC subsite with the total observed SPC cases. SPC subsites considered included oral cavity and pharynx, female genital system, male genital system, respiratory system, digestive system, breast, urinary system, brain, thyroid, lymphatic and hematopoietic diseases, skin excluding basal and squamous, and others. Observed cases of “others” were from those of subsites with a small number of observed cases and from those SPC subsites not mentioned above (SPC subsites considered)

HPV‐related head and neck cancer contributed more to the SIR of SPC after HPV‐related cancer than HPV‐related anogenital cancer (Table 1). In addition, how SIR was affected by SPC sites when IPC was limited to every single HPV‐related cancer was also provided (Tables S1‐S6). To better observe the distribution of SPC sites after every single initial primary HPV‐related cancer, we provided a figure, in which the percentage of the observed cases of every SPC site among the total observed SPC cases were presented (Figure 2). The commonest SPC sites were respiratory organs for initial primary oral (for both genders), anal cancer (for both genders), and cervical cancer, female genital organs for initial primary vulvar cancer, female genital organs and digestive organs for initial primary vaginal cancer, and male genital organs for initial primary penile cancer.

**TABLE 1:** SIRs of all sites after the diagnosis of HPV‐related cancer (initial primary sites shown)

	Males		Females	
Initial primary site	Observed	SIR (95% CI)	Observed	SIR (95% CI)
HPV‐related sites	3463	1.60 (1.55‐1.65)	7372	1.25 (1.22‐1.28)
HPV‐related oral cancer	2531	1.85 (1.78‐1.92)	911	2.25 (2.11‐2.40)
Vulva	NA	NA	1273	1.41 (1.34‐1.49)
Vagina	NA	NA	224	1.29 (1.13‐1.47)
Anus	454	1.22 (1.11‐1.33)	650	1.31 (1.21‐1.42)
Penis	478	1.13 (1.03‐1.24)	NA	NA
Cervix uteri	NA	NA	4314	1.10 (1.07‐1.13)

Abbreviations: 95% CI, 95% confidence interval; HPV, human papillomavirus; SIR, standardized incidence ratio; NA, not available.

**FIGURE 3:**
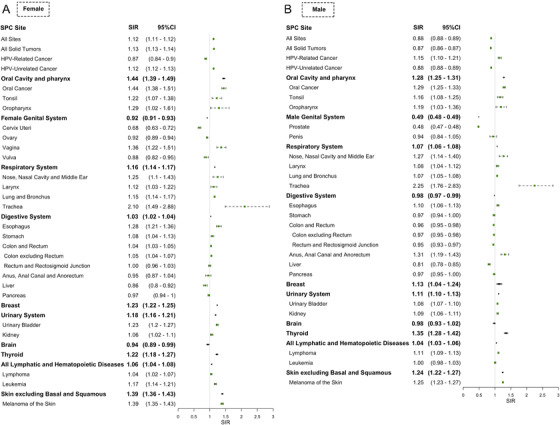
Subsite‐specific standardized incidence ratio (SIR) of second primary cancer (SPC) after the diagnosis of the initial primary human papillomavirus (HPV)‐unrelated cancer stratified by sex. Subsite‐specific SIR of developing SPC among survivors of initial primary HPV‐unrelated cancerwere shown. A, The SIR of second primary oral cavity and pharynx, genital system, respiratory system, digestive system, breast, urinary system, brain, thyroid, hematopoietic, and skin cancer after initial primary HPV‐unrelated cancer among females. B, The SIRs of second primary oral cavity and pharynx, genital system, respiratory system, digestive system, breast, urinary system, brain, thyroid, hematopoietic, and skin cancer after initial primary HPV‐unrelated cancer among men. The green dots represent SIR, and error bars represent 95% confidence interval. The vertical grey line indicates an SIR of 1.

SIR of SPC after HPV‐unrelated cancer (0.88 [95% CI, 0.88‐0.89] male and 1.12 [95% CI, 1.11‐1.12] female) was lower when compared with that of SPC after HPV‐related cancer (1.60 [95% CI, 1.55‐1.65] male and 1.25 [95% CI, 1.22‐1.28] female) (Figure 3).

**FIGURE 4:**
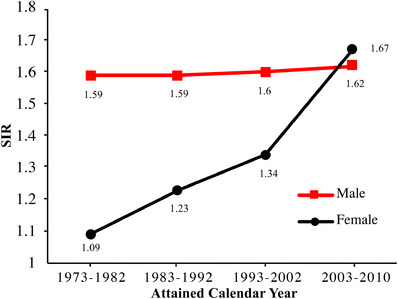
Standardized incidence ratio of second primary cancer after the diagnosis of the initial primary human papillomavirus (HPV)‐related cancer according to attained calendar year

The temporal pattern of SIR was apparent. SIR would be raised, if the IPC was diagnosed in more recent years (Figure 4). The 1‐, 3‐, 5‐, and 10‐year cumulative incidence of SPC was 1.19% (95% CI, 1.06‐1.33%) for male and 0.63% (95% CI, 0.57‐0.69%) for female, 4.78% (95% CI, 4.51‐5.06%) for male and 2.33% (95% CI, 2.22‐2.45%) for female, 7.22% (95% CI, 6.89‐7.55%) for male and 3.72% (95% CI, 3.58‐3.88%) for female, and 11.98% (95% CI, 11.56‐12.42%) for male and 6.87% (95% CI, 6.67‐7.08%) for female, respectively.

### 3.2 Risk factors of SPC

Marital status and TNM stage were significantly associated with SPC for both genders (Table 2). Age and surgery were also significant predictive factors of SPC for female patients.

**TABLE 2:** Hazard model of probabilities of SPC after initial primary HPV‐related cancer

Characteristic	SPC of males		SPC of females	
	sdHR (95% CI)	*P*‐value	sdHR (95% CI)	*P*‐value
Age				
18‐65 years	Reference			
65+ years	1.08 (0.92‐1.27)	.360	1.50 (1.28‐1.76)	<.001
Race				
White	Reference			
Black	1.04 (0.82‐1.32)	.730	1.08 (0.87‐1.34)	.470
Other/unknown	0.74 (0.51‐1.09)	.130	0.92 (0.72‐1.17)	.480
Marital status				
Married	Reference			
Unknown	0.77 (0.54‐1.10)	.150	0.88 (0.66‐1.17)	.390
Unmarried	0.74 (0.63‐0.86)	<.001	0.77 (0.66‐0.90)	<.001
TNM stage				
I	Reference			
II	0.74 (0.56‐1.00)	.049	1.11 (0.90‐1.36)	.320
III	0.87 (0.67‐1.15)	.330	0.84 (0.67‐1.05)	.120
IV	0.67 (0.53‐0.85)	.001	0.66 (0.50‐0.86)	.002
Unknown	0.67 (0.50‐0.91)	.009	1.10 (0.88‐1.37)	.420
Surgery				
Surgery performed	Reference			
Surgery not performed	0.97 (0.83‐1.13)	.660	0.73 (0.62‐0.86)	<.001
Unknown	0.98 (0.46‐2.11)	.960	0.14 (0.03‐0.55)	.005

Abbreviations: 95% CI, 95% confidence interval; sdHR, subdistribution hazard ratio; SPC, second primary cancer; TNM, tumor, node, and metastasis.

According to the result of subdistribution hazard regression, older female patients, with their IPC diagnosed when aged over 65 years old (subdistribution hazard ratio [sdHR] = 1.50 [95% CI, 1.28‐1.76], *P *< .001), were at more risk for SPC. As compared with married patients, those unmarried got a lower risk of developing SPC, with sdHR of 0.74 (95% CI, 0.63‐0.86, *P *< .001) for male and 0.77 (95% CI, 0.66‐0.90, *P *< .001) for female, respectively. In this study, those variables related to poor prognosis, including later TNM stage for both gender and surgery not performed for female, were also protective factors for SPC.

## 4 DISCUSSION

In this study, we estimated the risk and risk factors of SPC after the initial primary HPV‐related cancer. The SIR of SPC was 1.60 (95% CI, 1.55‐1.65) for male and 1.25 (95% CI, 1.22‐1.28) for female. The SIR, which was larger than 1, indicated that patients diagnosed with HPV‐related cancer were more likely to develop another primary cancer, as compared with the age‐specific reference population. Patients with the risk factors claimed in the competing risk analysis are suggested to screen for SPC regularly.

The elevation of SIR (>1) can partially be explained by their persistent carcinogenic lifestyle such as smoking and alcohol consumption.^40^, ^41^ In addition, patients once diagnosed with HPV‐related cancer may also be those who were genetically susceptible to cancer, thus being more likely to develop another primary cancer than the corresponding reference population.^42^ If those two hypotheses mentioned above were the only two explanations of SPC after HPV‐related cancer, we would have seen the similar SIR of different SPC sites, because the carcinogenic effect of both the carcinogenic lifestyle and being genetically susceptible to cancer exists in most of the cancers. However, we actually observed the elevation of SIR of SPC in HPV‐related sites, and this result is also supported by Neumann et al.^43^ Moreover, considering the strong carcinogenic effect of HPV in the HPV‐related sites,^6^, ^7^, ^8^, ^9^ it is suggested that some of the HPV‐related SPC cases may be caused by the persistent infection of HPV. In addition, patients with initial primary HPV‐related cancer may be those with a genetic predisposition to HPV infection, HPV transformation, and progression to HPV‐related cancer.^44^, ^45^, ^46^ This genetic susceptibility can increase the risk of second primary HPV‐related cancer due to the persistent infection of HPV in these patients and cause the elevated SIR, which is supported by the results of a meta‐analysis by Gilbert et al.^47^

HPV can be transmitted from the primary infected site to the later infected sites. To be specific, HPV virus can circulate to infect other organs, with HPV DNA and mRNA detected in peripheral blood of cervical cancer patients.^48^, ^49^ Another theory for the transmission of HPV from anus, cervix uteri, and other genital organs to oral cavity, oropharynx, and tonsil and then causing SPC is that HPV is transmitted in the process of oral sex or by touching the mouth with contaminated hands. Moreover, another primary cancer at the same site as that in IPC may also occur under the effect of HPV after the elimination of tumor cells of IPC. Next, in those organs infected later, a similar carcinogenic process of HPV as that in the IPC occurs. Considering the carcinogenic effect of HPV in both the IPC and its potential carcinogenic effect in second primary HPV‐related cancer, the HPV vaccine is promoted for the prevention of HPV infection, and thus HPV‐related IPC based on the benefit of HPV vaccination claimed by Nicol et al.^50^ However, this approved and currently utilized HPV vaccine is not intended for the elimination of HPV and transformed cells.^51^, ^52^ E6 and E7 proteins are necessary in the carcinogenic process of HPV and expressed by HPV‐transformed cells. Recently, there have been studies of therapeutic vaccines for established HPV infections and malignancies that target E6 and E7 proteins.^53^, ^54^ Therapeutic vaccines may be available in the future.

We observed the elevation of SIR in some of the potentially HPV‐related SPC sites including esophagus, larynx, nose and nasal sinus (for male only), lung and bronchus, trachea, and urinary bladder (for female only). Based on the hypothesis that the infection of HPV may be a reason for the occurrence of SPC, we think this may be indirect evidence for the carcinogenicity of HPV in these potentially HPV‐related sites, of which SIR was elevated. For instance, as was promoted by Xiong et al, HPV can be transmitted to and infect the lung by blood circulation, through the air or by high‐risk sexual behavior, through which HPV is transmitted to mouth, throat, and then lung.^55^ Then, HPV performs its carcinogenicity, causing second primary lung cancer.^48^, ^49^ This is also an indication of the necessity for further exploration of the carcinogenic effects of HPV in the esophagus, larynx, nose and nasal sinus, lung and bronchus, trachea, and urinary bladder.

The SIR of SPC after HPV‐unrelated cancer is lower than 1 for male. However, this is not evidence of that HPV‐unrelated cancer is a protective factor for the development of another primary cancer in male, because there have been many reports of SIR after HPV‐unrelated cancers such as hepatocellular cancer (10.07), stage IA nonsmall‐cell lung cancer (2.07), gastric cancer (1.11), and osteosarcoma (1.60), which was significantly larger than 1.^29^, ^56^, ^57^, ^58^ However, this can be an indication that there might be survivors from some specific cancers who may be less likely to develop another primary cancer when compared with the corresponding population due to the poor prognosis and other factors that need further investigation. For example, the SIR of SPC after the initial primary gastroenteropancreatic neuroendocrine tumors was reported to be 0.72.^59^

We also saw an increase of SIR by attained calendar year, which indicated a growing need for screening for SPC. Our results were similar to those by Suk et al.^60^ We assumed that the possible reason for the increase may be the improvement of survival from many of the HPV‐related cancers in recent years, and the development and popularization of the more effective detection approaches.^16^, ^17^, ^18^, ^19^, ^20^, ^21^, ^61^, ^62^ The improvement in survival reduced the competing effect of death with SPC, while the more effective detection approach made the detection of some cases possible. Future research concerning the reasons for the dramatic increase of SIR from 1993‐2002 to 2003‐2010 is necessary.

According to the results of the subdistribution hazard regression, some prognostic indicators of IPC were also indicators for the occurrence of SPC, with TNM stage (for both genders) and surgery (for females only) included. Further, indicators of better survival of the IPC also implied a larger chance of developing SPC, which was supported by Grundmann and Meyer.^25^

There are also some limitations in our study. First of all, although we believe in the quality of records in the SEER system, there may be some incorrect records of SPC in SEER due to the natural difficulty in distinguishing SPC from tumor metastasis and recurrence. Second, information about HPV infection and the lifestyle of patients such as smoking and alcohol consumption is not available in the SEER database. Because of the lack of information on HPV‐positivity status, HPV relatedness was by proxy in this study.

## 5 CONCLUSION

Patients diagnosed with HPV‐related cancer are more likely to develop another primary cancer, as compared with the age‐specific reference population. This study is of clinical significance and can help promote public health. According to our results, considering the persistent carcinogenesis of HPV and the SIR of SPC increasing by year, patients once diagnosed with HPV‐related cancer should screen for SPC regularly, especially when they have a high predictive risk for SPC according to the results of our subdistribution hazard regression. The observed distribution of SPC sites in this study should be taken into consideration, while making the plan of screening for SPC.

## CONFLICT OF INTEREST

The authors declare that there is no conflict of interest.

## AUTHOR CONTRIBUTIONS

Li Zhang, Huaqiang Zhou, Jiayi Shen, and Jiaqing Liu were responsible for the conception and design of the study, interpretation of data, drafting, and writing of the article. Other authors were responsible for interpretation of data and revision of the intellectual content. All the authors participated in final approval of the article and agreed to be accountable for all aspects of the work.

## Supporting information

Supporting Information

## Data Availability

The data that support the findings of this study are available in SEER Incidence Database at https://seer.cancer.gov/data/.
