# Supplementary material for: Incidence and risk factors of second primary cancer after the initial primary human papillomavirus related neoplasms
Source: MedComm (2020). 2020 Dec 3;1(3):400–9. doi: 10.1002/mco2.43 (PMC8491207; doi:10.1002/mco2.43)
Supplement: Supplementary file 1 — Supporting Information [file MCO2-1-400-s001.docx]

**Incidence and risk factors of second primary cancer after the initial primary human papillomavirus-related neoplasms**

**Contents:**

**Supplementary Tables**

**Table S1.** SIRs after the diagnosis of the initial primary HPV-related oral cancer.

**Table S2.** SIRs after the diagnosis of the initial primary anal cancer.

**Table S3.** SIRs after the diagnosis of the initial primary vulvar cancer.

**Table S4.** SIRs after the diagnosis of the initial primary vaginal cancer.

**Table S5.** SIRs after the diagnosis of the initial primary cervical cancer.

**Table S6.** SIRs after the diagnosis of the initial primary penile cancer.

Table S1. SIRs after the diagnosis of the initial primary HPV-related oral cancer

| **SPM Site** | **Male** | | **Female** | |
| --- | --- | --- | --- | --- |
|  | **Observed** | **SIR(95%CI)** | **Observed** | **SIR(95%CI)** |
| All Sites | 2,531 | 1.85^#^ (1.78-1.92) | 911 | 2.25^#^ (2.11-2.4) |
| All Solid Tumors | 2,332 | 1.91^#^ (1.83-1.98) | 831 | 2.30^#^ (2.15-2.47) |
| HPV-Related Cancer | 73 | 5.24^#^ (4.11-6.59) | 37 | 3.68^#^ (2.59-5.07) |
| HPV-Unrelated Cancer | 2,458 | 1.81^#^ (1.74-1.89) | 874 | 2.21^#^ (2.07-2.37) |
| **Oral Cavity and pharynx** | 508 | 11.42^#^ (10.44-12.45) | 192 | 28.02^#^ (24.19-32.27) |
| Oral Cancer | 353 | 13.94^#^ (12.53-15.47) | 136 | 31.35^#^ (26.30-37.08) |
| Tonsil | 36 | 4.95^#^ (3.47-6.85) | 17 | 26.81^#^ (15.62-42.93) |
| Oropharynx | 30 | 17.76^#^ (11.98-25.35) | 7 | 37.98^#^ (15.27-78.26) |
| **Female Genital System** | NA | NA | 37 | 0.77 (0.54-1.06) |
| Cervix Uteri | NA | NA | 5 | 1.10 (0.36-2.57) |
| Ovary | NA | NA | 11 | 0.84 (0.42-1.50) |
| Vagina | NA | NA | 2 | 2.69 (0.33-9.73) |
| Vulva | NA | NA | 5 | 2.09 (0.68-4.87) |
| **Male Genital System** | 433 | 0.99 (0.90-1.09) | NA | NA |
| Prostate | 431 | 1.00 (0.91-1.10) | NA | NA |
| Penis | 1 | 0.51 (0.01-2.85) | NA | NA |
| **Respiratory System** | 742 | 3.29^#^ (3.06-3.54) | 269 | 4.49^#^ (3.97-5.06) |
| Nose, Nasal Cavity and Middle Ear | 14 | 6.78^#^ (3.70-11.37) | 1 | 1.93 (0.05-10.74) |
| Larynx | 69 | 3.78^#^ (2.94-4.79) | 22 | 14.34^#^ (8.99-21.72) |
| Lung and Bronchus | 656 | 3.21^#^ (2.97-3.46) | 244 | 4.23^#^ (3.71-4.79) |
| Trachea | 3 | 13.32^#^ (2.75-38.94) | 2 | 36.97^#^ (4.48-133.54) |
| **Digestive System** | 407 | 1.53^#^ (1.38-1.68) | 175 | 2.11^#^ (1.81-2.44) |
| Esophagus | 122 | 5.76^#^ (4.78-6.88) | 52 | 21.26^#^ (15.88-27.88) |
| Stomach | 42 | 1.57^#^ (1.13-2.12) | 8 | 1.32 (0.57-2.61) |
| Colon and Rectum | 139 | 1.00 (0.84-1.18) | 73 | 1.45^#^ (1.14-1.83) |
| Colon excluding Rectum | 92 | 0.96 (0.78-1.18) | 56 | 1.46^#^ (1.10-1.89) |
| Rectum and Rectosigmoid Junction | 47 | 1.08 (0.80-1.44) | 17 | 1.44 (0.84-2.31) |
| Anus, Anal Canal and Anorectum | 6 | 1.99 (0.73-4.33) | 1 | 0.64 (0.02-3.57) |
| Liver | 37 | 1.59^#^ (1.12-2.19) | 11 | 3.99^#^ (1.99-7.14) |
| Pancreas | 44 | 1.24 (0.90-1.67) | 19 | 1.51 (0.91-2.36) |
| **Breast** | 1 | 0.34 (0.01-1.92) | 115 | 0.98 (0.81-1.18) |
| **Urinary System** | 138 | 0.96 (0.80-1.13) | 21 | 1.02 (0.63-1.56) |
| Urinary Bladder | 82 | 0.87 (0.70-1.08) | 13 | 1.17 (0.62-2.01) |
| Kidney | 54 | 1.25 (0.94-1.63) | 6 | 0.76 (0.28-1.65) |
| **Brain** | 11 | 0.76 (0.38-1.35) | 2 | 0.51 (0.06-1.84) |
| **Thyroid** | 13 | 1.25 (0.67-2.14) | 7 | 1.10 (0.44-2.27) |
| **All Lymphatic and Hematopoietic Diseases** | 147 | 1.27^#^ (1.07-1.49) | 55 | 1.68^#^ (1.26-2.19) |
| Lymphoma | 84 | 1.47^#^ (1.17-1.82) | 36 | 2.10^#^ (1.47-2.91) |
| Leukemia | 39 | 0.99 (0.70-1.36) | 15 | 1.50 (0.84-2.48) |
| **Skin excluding Basal and Squamous** | 60 | 0.92 (0.70-1.18) | 11 | 0.91 (0.45-1.62) |
| Melanoma of the Skin | 51 | 0.85 (0.63-1.12) | 8 | 0.73 (0.32-1.44) |

Confidence intervals are 95%.

^#^ P<0.05.

^*^ Confidence interval.

Table S2. SIRs after the diagnosis of the initial primary anal cancer

| **SPM Site** | **Male** | | **Female** | |
| --- | --- | --- | --- | --- |
|  | **Observed** | **SIR(95%CI^*^)** | **Observed** | **SIR(95%CI^*^)** |
| All Sites | 454 | 1.22^#^ (1.11-1.33) | 650 | 1.31^#^ (1.21-1.42) |
| All Solid Tumors | 403 | 1.21^#^ (1.10-1.34) | 588 | 1.34^#^ (1.23-1.45) |
| HPV-Related Cancer | 34 | 10.15^#^ (7.03-14.18) | 59 | 4.96^#^ (3.78-6.40) |
| HPV-Unrelated Cancer | 420 | 1.14^#^ (1.03-1.25) | 591 | 1.22^#^ (1.12-1.32) |
| **Oral Cavity and pharynx** | 23 | 2.08^#^ (1.32-3.12) | 16 | 1.96^#^ (1.12-3.18) |
| Oral Cancer | 13 | 2.04^#^ (1.09-3.49) | 10 | 1.89 (0.91-3.48) |
| Tonsil | 5 | 3.13^#^ (1.01-7.29) | 2 | 2.83 (0.34-10.21) |
| Oropharynx | 0 | 0 (0-9.16) | 0 | 0 (0-17.24) |
| **Female Genital System** | NA | NA | 96 | 1.70^#^ (1.38-2.08) |
| Cervix Uteri | NA | NA | 5 | 1.03 (0.34-2.41) |
| Ovary | NA | NA | 14 | 0.90 (0.49-1.51) |
| Vagina | NA | NA | 8 | 8.46^#^ (3.65-16.68) |
| Vulva | NA | NA | 24 | 7.50^#^ (4.81-11.16) |
| **Male Genital System** | 65 | 0.56^#^ (0.43-0.71) | NA | NA |
| Prostate | 63 | 0.55^#^ (0.42-0.7) | NA | NA |
| Penis | 0 | 0 (0-6.63) | NA | NA |
| **Respiratory System** | 128 | 2.03^#^ (1.69-2.41) | 162 | 2.25^#^ (1.91-2.62) |
| Nose, Nasal Cavity and Middle Ear | 0 | 0 (0-6.81) | 3 | 4.66 (0.96-13.63) |
| Larynx | 12 | 2.50^#^ (1.29-4.37) | 2 | 1.18 (0.14-4.27) |
| Lung and Bronchus | 116 | 2.01^#^ (1.66-2.42) | 157 | 2.25^#^ (1.91-2.63) |
| Trachea | 0 | 0 (0-61.52) | 0 | 0 (0-58.12) |
| **Digestive System** | 108 | 1.46^#^ (1.20-1.77) | 128 | 1.20^#^ (1.00-1.43) |
| Esophagus | 7 | 1.26 (0.51-2.60) | 5 | 1.65 (0.53-3.84) |
| Stomach | 4 | 0.53 (0.14-1.35) | 12 | 1.57 (0.81-2.75) |
| Colon and Rectum | 46 | 1.16 (0.85-1.54) | 66 | 1.02 (0.79-1.30) |
| Colon excluding Rectum | 25 | 0.90 (0.58-1.32) | 47 | 0.94 (0.69-1.25) |
| Rectum and Rectosigmoid Junction | 21 | 1.78^#^ (1.10-2.72) | 19 | 1.32 (0.79-2.06) |
| Anus, Anal Canal and Anorectum | 29 | 36.57^#^ (24.49-52.52) | 20 | 10.08^#^ (6.16-15.57) |
| Liver | 6 | 1.09 (0.40-2.37) | 4 | 1.20 (0.33-3.07) |
| Pancreas | 8 | 0.82 (0.35-1.62) | 16 | 0.98 (0.56-1.59) |
| **Breast** | 2 | 2.52 (0.31-9.10) | 120 | 0.87 (0.72-1.04) |
| **Urinary System** | 47 | 1.17 (0.86-1.55) | 32 | 1.22 (0.84-1.72) |
| Urinary Bladder | 35 | 1.28 (0.89-1.79) | 24 | 1.67^#^ (1.07-2.48) |
| Kidney | 9 | 0.82 (0.37-1.55) | 7 | 0.72 (0.29-1.47) |
| **Brain** | 2 | 0.53 (0.06-1.92) | 5 | 1.07 (0.35-2.49) |
| **Thyroid** | 2 | 0.84 (0.10-3.02) | 5 | 0.66 (0.21-1.54) |
| **All Lymphatic and Hematopoietic Diseases** | 39 | 1.21 (0.86-1.66) | 41 | 0.98 (0.70-1.33) |
| Lymphoma | 28 | 1.79^#^ (1.19-2.59) | 20 | 0.93 (0.57-1.43) |
| Leukemia | 9 | 0.81 (0.37-1.54) | 16 | 1.22 (0.70-1.98) |
| **Skin excluding Basal and Squamous** | 19 | 1.12 (0.67-1.75) | 16 | 1.03 (0.59-1.68) |
| Melanoma of the Skin | 13 | 0.85 (0.45-1.45) | 16 | 1.15 (0.66-1.87) |

Confidence intervals are 95%.

^#^ P<0.05.

^*^ Confidence interval.

Table S3. SIRs after the diagnosis of the initial primary vulvar cancer

| **SPM Site** | **Observed** | **Expected** | **SIR** | **95%CI^*^** |
| --- | --- | --- | --- | --- |
| All Sites | 1,273 | 901.26 | 1.41^#^ | 1.34-1.49 |
| All Solid Tumors | 1,160 | 792.33 | 1.46^#^ | 1.38-1.55 |
| HPV-Related Cancer | 288 | 21.82 | 13.20^#^ | 11.72-14.82 |
| HPV-Unrelated Cancer | 985 | 879.45 | 1.12^#^ | 1.05-1.19 |
| **Oral Cavity and pharynx** | 29 | 14.85 | 1.95^#^ | 1.31-2.80 |
| Oral Cavity | 22 | 9.79 | 2.25^#^ | 1.41-3.40 |
| Tonsil | 2 | 1.16 | 1.73 | 0.21-6.25 |
| Oropharynx | 0 | 0.36 | 0 | 0-10.12 |
| **Female Genital System** | 339 | 98.71 | 3.43^#^ | 3.08-3.82 |
| Cervix Uteri | 14 | 8.94 | 1.57 | 0.86-2.63 |
| Ovary | 27 | 28.15 | 0.96 | 0.63-1.40 |
| Vagina | 28 | 1.77 | 15.85^#^ | 10.53-22.91 |
| Vulva | 207 | 6.21 | 33.31^#^ | 28.93-38.18 |
| **Male Genital System** | NA | NA | NA | NA |
| Prostate | NA | NA | NA | NA |
| Penis | NA | NA | NA | NA |
| **Respiratory System** | 215 | 125.88 | 1.71^#^ | 1.49-1.95 |
| Nose, Nasal Cavity and Middle Ear | 2 | 1.20 | 1.67 | 0.20-6.02 |
| Larynx | 10 | 2.84 | 3.52^#^ | 1.69-6.48 |
| Lung and Bronchus | 202 | 121.57 | 1.66^#^ | 1.44-1.91 |
| Trachea | 1 | 0.11 | 8.70 | 0.22-48.49 |
| **Digestive System** | 244 | 206.55 | 1.18^#^ | 1.04-1.34 |
| Esophagus | 7 | 5.64 | 1.24 | 0.50-2.56 |
| Stomach | 13 | 15.15 | 0.86 | 0.46-1.47 |
| Colon and Rectum | 131 | 127.73 | 1.03 | 0.86-1.22 |
| Colon excluding Rectum | 100 | 99.93 | 1.00 | 0.81-1.22 |
| Rectum and Rectosigmoid Junction | 31 | 27.81 | 1.11 | 0.76-1.58 |
| Anus, Anal Canal and Anorectum | 37 | 3.38 | 10.95^#^ | 7.71-15.10 |
| Liver | 5 | 5.92 | 0.84 | 0.27-1.97 |
| Pancreas | 30 | 30.92 | 0.97 | 0.65-1.39 |
| **Breast** | 219 | 244.25 | 0.90 | 0.78-1.02 |
| **Urinary System** | 54 | 48.27 | 1.12 | 0.84-1.46 |
| Urinary Bladder | 30 | 27.53 | 1.09 | 0.74-1.56 |
| Kidney | 16 | 16.85 | 0.95 | 0.54-1.54 |
| **Brain** | 9 | 8.33 | 1.08 | 0.49-2.05 |
| **Thyroid** | 10 | 12.31 | 0.81 | 0.39-1.49 |
| **All Lymphatic and Hematopoietic Diseases** | 73 | 77.68 | 0.94 | 0.74-1.18 |
| Lymphoma | 40 | 39.75 | 1.01 | 0.72-1.37 |
| Leukemia | 16 | 25.14 | 0.64 | 0.36-1.03 |
| **Skin excluding Basal and Squamous** | 39 | 27.28 | 1.43^#^ | 1.02-1.95 |
| Melanoma of the Skin | 32 | 24.21 | 1.32 | 0.90-1.87 |

Confidence intervals are 95%.

^#^ P<0.05.

^*^ Confidence interval.

Table S4. SIRs after the diagnosis of the initial primary vaginal cancer

| **SPM Site** | **Observed** | **Expected** | **SIR** | **95%CI^*^** |
| --- | --- | --- | --- | --- |
| All Sites | 224 | 173.58 | 1.29^#^ | 1.13-1.47 |
| All Solid Tumors | 201 | 153.36 | 1.31^#^ | 1.14-1.50 |
| HPV-Related Cancer | 35 | 4.56 | 7.68^#^ | 5.35-10.68 |
| HPV-Unrelated Cancer | 189 | 169.03 | 1.12 | 0.96-1.29 |
| **Oral Cavity and pharynx** | 2 | 2.82 | 0.71 | 0.09-2.56 |
| Oral Cavity | 1 | 1.81 | 0.55 | 0.01-3.08 |
| Tonsil | 0 | 0.24 | 0 | 0-15.20 |
| Oropharynx | 0 | 0.07 | 0 | 0-50.86 |
| **Female Genital System** | 48 | 19.90 | 2.41^#^ | 1.78-3.20 |
| Cervix Uteri | 5 | 2.16 | 2.32 | 0.75-5.41 |
| Ovary | 6 | 5.48 | 1.10 | 0.40-2.38 |
| Vagina | 11 | 0.36 | 30.48^#^ | 15.21-54.53 |
| Vulva | 17 | 1.08 | 15.70^#^ | 9.15-25.14 |
| **Male Genital System** | NA | NA | NA | NA |
| Prostate | NA | NA | NA | NA |
| Penis | NA | NA | NA | NA |
| **Respiratory System** | 38 | 24.20 | 1.57^#^ | 1.11-2.16 |
| Nose, Nasal Cavity and Middle Ear | 0 | 0.23 | 0 | 0-16.00 |
| Larynx | 1 | 0.61 | 1.64 | 0.04-9.12 |
| Lung and Bronchus | 37 | 23.30 | 1.59^#^ | 1.12-2.19 |
| Trachea | 0 | 0.02 | 0 | 0-160.02 |
| **Digestive System** | 48 | 39.46 | 1.22 | 0.90-1.61 |
| Esophagus | 5 | 1.12 | 4.46^#^ | 1.45-10.42 |
| Stomach | 4 | 3.14 | 1.27 | 0.35-3.26 |
| Colon and Rectum | 25 | 24.12 | 1.04 | 0.67-1.53 |
| Colon excluding Rectum | 15 | 18.74 | 0.80 | 0.45-1.32 |
| Rectum and Rectosigmoid Junction | 10 | 5.37 | 1.86 | 0.89-3.42 |
| Anus, Anal Canal and Anorectum | 2 | 0.64 | 3.11 | 0.38-11.25 |
| Liver | 2 | 1.19 | 1.68 | 0.20-6.07 |
| Pancreas | 4 | 5.94 | 0.67 | 0.18-1.73 |
| **Breast** | 39 | 47.95 | 0.81 | 0.58-1.11 |
| **Urinary System** | 16 | 8.98 | 1.78^#^ | 1.02-2.89 |
| Urinary Bladder | 12 | 5.01 | 2.40^#^ | 1.24-4.19 |
| Kidney | 2 | 3.27 | 0.61 | 0.07-2.21 |
| **Brain** | 0 | 1.58 | 0 | 0-2.33 |
| **Thyroid** | 2 | 2.47 | 0.81 | 0.10-2.92 |
| **All Lymphatic and Hematopoietic Diseases** | 15 | 14.43 | 1.04 | 0.58-1.71 |
| Lymphoma | 7 | 7.20 | 0.97 | 0.39-2.00 |
| Leukemia | 5 | 4.54 | 1.10 | 0.36-2.57 |
| **Skin excluding Basal and Squamous** | 7 | 4.80 | 1.46 | 0.59-3.00 |
| Melanoma of the Skin | 6 | 4.29 | 1.40 | 0.51-3.05 |

Confidence intervals are 95%.

^#^ P<0.05.

^*^ Confidence interval.

Table S5. SIRs after the diagnosis of the initial primary cervical cancer

| **SPM Site** | **Observed** | **Expected** | **SIR** | **95%CI^*^** |
| --- | --- | --- | --- | --- |
| All Sites | 4,314 | 3922.57 | 1.10^#^ | 1.07-1.13 |
| All Solid Tumors | 3,858 | 3529.96 | 1.09^#^ | 1.06-1.13 |
| HPV-Related Cancer | 316 | 121.00 | 2.62^#^ | 2.34-2.92 |
| HPV-Unrelated Cancer | 3,998 | 3,802.00 | 1.05^#^ | 1.02-1.08 |
| **Oral Cavity and pharynx** | 88 | 64.73 | 1.36^#^ | 1.09-1.68 |
| Oral Cavity | 47 | 39.23 | 1.20 | 0.88-1.59 |
| Tonsil | 17 | 6.55 | 2.60^#^ | 1.51-4.16 |
| Oropharynx | 3 | 1.73 | 1.73 | 0.36-5.06 |
| **Female Genital System** | 506 | 499.83 | 1.01 | 0.93-1.10 |
| Cervix Uteri | 68 | 68.02 | 1.00 | 0.78-1.27 |
| Ovary | 125 | 130.39 | 0.96 | 0.80-1.14 |
| Vagina | 117 | 7.17 | 16.33^#^ | 13.50-19.57 |
| Vulva | 77 | 21.58 | 3.57^#^ | 2.82-4.46 |
| **Male Genital System** | NA | NA | NA | NA |
| Prostate | NA | NA | NA | NA |
| Penis | NA | NA | NA | NA |
| **Respiratory System** | 993 | 516.56 | 1.92^#^ | 1.80-2.05 |
| Nose, Nasal Cavity and Middle Ear | 7 | 4.95 | 1.41 | 0.57-2.91 |
| Larynx | 30 | 15.00 | 2.00^#^ | 1.35-2.86 |
| Lung and Bronchus | 954 | 495.44 | 1.93^#^ | 1.81-2.05 |
| Trachea | 2 | 0.52 | 3.88 | 0.47-14.01 |
| **Digestive System** | 883 | 727.21 | 1.21^#^ | 1.14-1.30 |
| Esophagus | 28 | 21.44 | 1.31 | 0.87-1.89 |
| Stomach | 86 | 56.93 | 1.51^#^ | 1.21-1.87 |
| Colon and Rectum | 519 | 432.04 | 1.20^#^ | 1.10-1.31 |
| Colon excluding Rectum | 349 | 323.63 | 1.08 | 0.97-1.20 |
| Rectum and Rectosigmoid Junction | 170 | 108.41 | 1.57^#^ | 1.34-1.82 |
| Anus, Anal Canal and Anorectum | 34 | 15.77 | 2.16^#^ | 1.49-3.01 |
| Liver | 25 | 27.17 | 0.92 | 0.60-1.36 |
| Pancreas | 107 | 107.26 | 1.00 | 0.82-1.21 |
| **Breast** | 847 | 1241.22 | 0.68^#^ | 0.64-0.73 |
| **Urinary System** | 315 | 179.65 | 1.75^#^ | 1.57-1.96 |
| Urinary Bladder | 212 | 90.14 | 2.35^#^ | 2.05-2.69 |
| Kidney | 73 | 77.09 | 0.95 | 0.74-1.19 |
| **Brain** | 29 | 38.67 | 0.75 | 0.50-1.08 |
| **Thyroid** | 72 | 96.00 | 0.75^#^ | 0.59-0.94 |
| **All Lymphatic and Hematopoietic Diseases** | 289 | 295.99 | 0.98 | 0.87-1.10 |
| Lymphoma | 165 | 156.74 | 1.05 | 0.90-1.23 |
| Leukemia | 93 | 86.92 | 1.07 | 0.86-1.31 |
| **Skin excluding Basal and Squamous** | 80 | 136.11 | 0.59^#^ | 0.47-0.73 |
| Melanoma of the Skin | 70 | 124.98 | 0.56^#^ | 0.44-0.71 |

Confidence intervals are 95%.

^#^ P<0.05.

^*^ Confidence interval.

Table S6. SIRs after the diagnosis of the initial primary penile cancer

| **SPM Site** | **Observed** | **Expected** | **SIR** | **95%CI^*^** |
| --- | --- | --- | --- | --- |
| All Sites | 478 | 421.56 | 1.13^#^ | 1.03-1.24 |
| All Solid Tumors | 419 | 374.60 | 1.12^#^ | 1.01-1.23 |
| HPV-Related Cancer | 43 | 3.01 | 14.31^#^ | 10.35-19.27 |
| HPV-Unrelated Cancer | 435 | 418.56 | 1.04 | 0.94-1.14 |
| **Oral Cavity and pharynx** | 21 | 11.33 | 1.85^#^ | 1.15-2.83 |
| Oral Cavity | 13 | 6.74 | 1.93^#^ | 1.03-3.30 |
| Tonsil | 2 | 1.25 | 1.59 | 0.19-5.76 |
| Oropharynx | 0 | 0.36 | 0 | 0-10.29 |
| **Female Genital System** | NA | NA | NA | NA |
| Cervix Uteri | NA | NA | NA | NA |
| Ovary | NA | NA | NA | NA |
| Vagina | NA | NA | NA | NA |
| Vulva | NA | NA | NA | NA |
| **Male Genital System** | 149 | 128.99 | 1.16 | 0.98-1.36 |
| Prostate | 108 | 127.62 | 0.85 | 0.69-1.02 |
| Penis | 37 | 0.68 | 54.41^#^ | 38.31-75.00 |
| **Respiratory System** | 117 | 74.62 | 1.57^#^ | 1.30-1.88 |
| Nose, Nasal Cavity and Middle Ear | 0 | 0.60 | 0 | 0-6.15 |
| Larynx | 10 | 5.30 | 1.89 | 0.91-3.47 |
| Lung and Bronchus | 107 | 68.55 | 1.56^#^ | 1.28-1.89 |
| Trachea | 0 | 0.07 | 0 | 0-51.89 |
| **Digestive System** | 81 | 87.59 | 0.92 | 0.73-1.15 |
| Esophagus | 7 | 5.98 | 1.17 | 0.47-2.41 |
| Stomach | 10 | 9.64 | 1.04 | 0.50-1.91 |
| Colon and Rectum | 42 | 49.61 | 0.85 | 0.61-1.14 |
| Colon excluding Rectum | 28 | 35.70 | 0.78 | 0.52-1.13 |
| Rectum and Rectosigmoid Junction | 14 | 13.91 | 1.01 | 0.55-1.69 |
| Anus, Anal Canal and Anorectum | 4 | 0.71 | 5.61^#^ | 1.53-14.37 |
| Liver | 5 | 4.96 | 1.01 | 0.33-2.35 |
| Pancreas | 9 | 11.17 | 0.81 | 0.37-1.53 |
| **Breast** | 0 | 0.88 | 0 | 0-4.20 |
| **Urinary System** | 31 | 45.51 | 0.68^#^ | 0.46-0.97 |
| Urinary Bladder | 22 | 32.47 | 0.68 | 0.42-1.03 |
| Kidney | 5 | 10.59 | 0.47 | 0.15-1.10 |
| **Brain** | 0 | 3.80 | 0^#^ | 0-0.97 |
| **Thyroid** | 3 | 1.89 | 1.58 | 0.33-4.63 |
| **All Lymphatic and Hematopoietic Diseases** | 42 | 35.30 | 1.19 | 0.86-1.61 |
| Lymphoma | 20 | 16.38 | 1.22 | 0.75-1.89 |
| Leukemia | 20 | 12.84 | 1.56 | 0.95-2.41 |
| **Skin excluding Basal and Squamous** | 15 | 16.12 | 0.93 | 0.52-1.54 |
| Melanoma of the Skin | 11 | 14.40 | 0.76 | 0.38-1.37 |

Confidence intervals are 95%.

^#^ P<0.05.

^*^ Confidence interval.
